# Evaluation of the National Comprehensive Cancer Network and European Society for Medical Oncology Nasopharyngeal Carcinoma Surveillance Guidelines

**DOI:** 10.3389/fonc.2020.00119

**Published:** 2020-02-14

**Authors:** Guan-Qun Zhou, Jia-Wei Lv, Ling-long Tang, Yan-Ping Mao, Rui Guo, Jun Ma, Ying Sun

**Affiliations:** State Key Laboratory of Oncology in Southern China, Department of Radiation Oncology, Collaborative Innovation Center of Cancer Medicine, Cancer Center, Sun Yat-sen University, Guangzhou, China

**Keywords:** national comprehensive cancer network, European Society for Medical Oncology, guidelines, surveillance, nasopharyngeal carcinoma

## Abstract

**Purpose:** The National Comprehensive Cancer Network (NCCN) and European Society for Medical Oncology (ESMO) provide surveillance guidelines for nasopharyngeal carcinoma (NPC). We evaluated the ability of these guidelines to capture disease recurrence.

**Materials and methods:** All 749 NPC patients were stratified for analysis by T and N stage. We evaluated the guidelines by calculating the percentage of relapses detected when following the 2018 NCCN, 2015 NCCN, and 2012 ESMO surveillance guidelines, and related surveillance costs were compared.

**Results:** At a median follow-up of 100.8 months, 168 patients (22.4%) had experienced recurrence. Nineteen recurrences (11.3%) were detected using the 2018 NCCN, 53 (31.5%) using the 2015 NCCN and 46 (27.4%) using the ESMO guidelines. To capture 95% recurrences, surveillance would be required for 85.57 months for T1/2, 67.45 months for T3/4, 83.57 months for N0/1, and 55.80 months for N2/3 disease. In T1/2 disease, Medicare surveillance costs per patient were US$1642.66 using 2018 NCCN or ESMO and US$2179.81 using 2015 NCCN. Costs per recurrence detected were US$42,578.64, 62,088.70, and 73,329.76 using 2018 NCCN, 2015 NCCN, and ESMO, respectively.

**Conclusions:** If strictly followed, the NCCN and ESMO guidelines will miss more than two-thirds recurrences. Improved surveillance algorithms to balance patient benefit against costs are needed.

## Introduction

Nasopharyngeal carcinoma (NPC) is radiosensitive and radiation was the mainstay definitive treatment. Though excellent control especially in local and regional disease can be achieved, recurrence after primary treatment is a major threat for NPC patients, particularly in patients who present with advanced stage NPC. Close follow-up can accurately assess treatment response as well as early detect the recurrent disease, and it can salvage a percentage of patients amenable to radical surgery or re-irradiation (1). However, intensive review can also incur considerable costs.

Despite an evident necessity, the optimal follow-up schedule and regimen for NPC patients after radical radiotherapy has not been thoroughly addressed. The National Comprehensive Cancer Network (NCCN) and European Society for Medical Oncology (ESMO) provide well-recognized follow-up guidelines for (2–4). However, these recommended surveillance protocols for NPC were somewhat contradicted. In the past many years, the NCCN recommended annual magnetic resonance imaging (MRI) for T3/4 or N2/3 disease due to the inaccessibility of the nasopharynx. In 2018, the NCCN updated their recommendations and suggested neither routine imaging of the nasopharynx nor the neck in patients without signs or symptoms, in view of the fact that in most cases recurrence is reported by patients themselves. Despite this change, the NCCN and the ESMO protocols are not uniform. Due to lack of prospective randomized data, there is no definitive evidence to clarify which regimen is most effective. As a result, there is significant heterogeneity in the follow-up strategies developed by different clinicians, leading to over- and underutilization of surveillance in certain patient populations (5). This variability of health care may translate into a unreasonable allocation of medical resources.

In the present study, we sought to evaluate the performance of the 2018 NCCN, 2015 NCCN, and 2012 ESMO guidelines by calculating how many NPC relapses could be detected when patients follow the surveillance recommendations of these guidelines. After that we calculated the duration of continuous monitoring at different sites in patients with different stages of NPC in order to detect 90, 95, and 100% of recurrent events. Finally, the average cost per recurrent event was compared for follow-up according to the guidelines and assumptions to detect 95% of recurrent events.

## Patients and Methods

### Patient Population

After obtaining approval from the institutional review board of Sun Yat-sen University Cancer Center, we prospectively reviewed our NPC registry system, and identified 778 patients treated with radical intensity modulated radiation therapy (IMRT) or combined chemoradiotherapy for newly diagnosed, non-metastatic NPC between January 2003 and December 2010 at our Cancer Center. Written informed consent was obtained from each patient for their information to be used in research without affecting their treatment options or violating their privacy informed consent was obtained from the participants of this study. If the participants were under the age of 16, written informed consent was obtained from the parents or guardians of participants.

### Treatment

All patients received radical IMRT for the entire course of treatment. Details regarding the IMRT techniques have been reported in a previous study (6). During the study period, the therapeutic principles in our institution recommended radiotherapy alone for NPC patients with stage I disease, concurrent chemoradiotherapy for patients with stage II, and concurrent chemoradiotherapy with or without neoadjuvant/adjuvant chemotherapy for stage III–IVb. If necessary, salvage treatments including brachytherapy, surgery, and chemotherapy, were provided in the cases of relapse or persistent disease.

### Follow-Up

Because of the retrospective nature, the actual follow up interval and items of this study were not standardized. However, most patients underwent history and physical examination every 3 months for the first 2 years, every 6 months for up to 5 years and then annually. Post-treatment baseline MRI of the nasopharynx and neck within 3 months after treatment was compulsory. Nasopharyngoscopy, MRI of the nasopharynx and neck, chest radiography, or computed tomography (CT), abdominal ultrasonography or CT and whole-body skeletal scintigraphy were recommended to be performed annually or if clinically indicated by tumor recurrence.

### Classification of Disease Recurrence

Recurrence disease was defined as relapse tumor at the primary site, regional lymph nodes, or distant sites that was radiographically or pathologically confirmed at least 30 days after treatment. Recurrences were classified by site as nasopharynx, neck, bone, chest, abdomen, or other sites. Using the location categories described above, it is possible to directly translate into the type of imaging or clinical examinations required for follow-up in each site. The first recurrence in each patient was counted as an event and all other recurrences were censored to avoid double counting. Recurrence that occurs simultaneously at multiple sites was individually counted.

### Evaluation of Current Guidelines

Table 1 lists the recommended surveillance regimen according to the 2018 NCCN, 2015 NCCN, and 2012 ESMO guidelines. The ability of the guidelines was evaluated by calculating the total recurrences events that would be detected if patients were followed up strictly according to the strategies recommended by the guidelines. Because recurrences in the neck can be detected clinically or via imaging of the neck, the detection of recurrences in the neck was based on the time point recommended for physical examination; detection of recurrences in the nasopharynx, bone, chest and abdomen were via imaging of the nasopharynx, bone, chest or abdomen, respectively. To evaluate the guidelines, patients were stratified according to T and N classification (T1/2 vs. T3/4; N0/1 vs. N2/3). All patients were restaged according to the 7th edition of the International Union Against Cancer/American Joint Committee on Cancer system (7).

**Table 1:** NCCN, ESMO, and AHNS oncologic surveillance schedules for NPC.

	**Year 1**	**Year 2**	**Year 3**	**Year 4**	**Year 5**	**>5 years**
**2018 NCCN**						
H&P exam	1–3 months	2–6 months	4–8 months	4–8 months	4–8 months	12 months
EBV serology	Consider EBV DNA monitoring					
Baseline imaging	Not routinely recommended					
Chest imaging	Chest CT with or without contrast as clinically indicated for patients with smoking history					
Abdominal imaging	Not mentioned					
Bone scan	Not mentioned					
**2015 NCCN**						
H&P exam	1–3 months	2–6 months	4–8 months	4–8 months	4–8 months	12 months
EBV serology	Consider EBV DNA monitoring					
Baseline imaging	Annual for T3–4 or N2–3 disease only					
Chest imaging	Annual low-dose chest CT for patients with high risk of lung cancer^#^					
Abdominal imaging	Not mentioned					
Bone scan	Not mentioned					
**2012 ESMO**						
H&P exam	Periodic examination of the nasopharynx and neck, cranial nerve function					
EBV serology	Post-treatment plasma/serum load of EBV DNA					
Baseline imaging	Used on a 6- to 12-month basis for the first few years for T3 and T4 tumors					
Chest imaging	Not mentioned					
Abdominal imaging	Not mentioned					
Bone scan	Not mentioned					

*NCCN, National Comprehensive Cancer Network; ESMO, European Society for Medical Oncology; EBV, Epstein -Barr viral*.

# *Refer to patients aged 55–74 years and >30 pack-year history of smoking and smoking cessation <15 y or patients aged >50 years and >20 pack-year history of smoking and one additional risk factor (other than second-hand smoke) according to NCCN Guidelines Version 1. 2016 Lung Cancer Screening*.

Both the 2018 and 2015 NCCN guidelines use a time range (e.g., 1–3 months) for the frequency of the history and physical examination, which are too vague for evaluation and comparison; we used the median of the recommended time range (e.g., 2 months for 1–3 months). The 2012 ESMO guidelines suggest periodic history and physical examination; we used the frequency suggested by the 2015 NCCN. The ESMO guidelines suggest nasopharyngeal MRI on a 6–12 months basis for the first few years for T3 and T4 tumors; we specified this as nasopharyngeal MRI every 9 months for the first 5 years.

### Medicare Cost Analysis

Using charges issued in 2017 by the Medical Insurance Administration Bureau of Guangzhou, China, the surveillance costs were estimated on a per-patient basis when the recommended follow-up schedules were strictly adhered to and completed, as shown in Table 1. The Chinese currency was converted to US dollars based on exchange rate and date [US$1.00 = ¥6.75 [¥ being the Chinese currency in 2017]]. The cost of capturing 95% recurrences was based on the following estimates: the frequency of the history and physical examination was similar to that recommended in the 2018 and 2015 guidelines; and including annual head and neck MRI, annual skeletal scintigraphy, annual chest CT, and annual abdominal CT. Finally, the cost of detecting one recurrent case in each stage group was calculated.

### Statistical Analysis

The duration of follow-up required to find 90, 95, and 100% of recurrences at each location by stage stratification was determined by the cumulative frequency of time to relapse. For subgroups that the follow-up time required to detect 95% of recurrent could not be calculated for too few events, it was estimated to be half-way between the time for capturing 90 and 100%. Recurrence rates after treatment were estimated using the Kaplan–Meier and differences were calculated using log–rank tests. The required surveillance durations for the different stages of disease were compared using the Mann–Whitney *U*-test. All tests were two-sided, with *P* < 0.05 considered significant. Statistical analysis was performed using SPSS version 19.0 (IBM, Armonk, NY, USA).

## Results

### Patient Demographics

**Figure 1:**
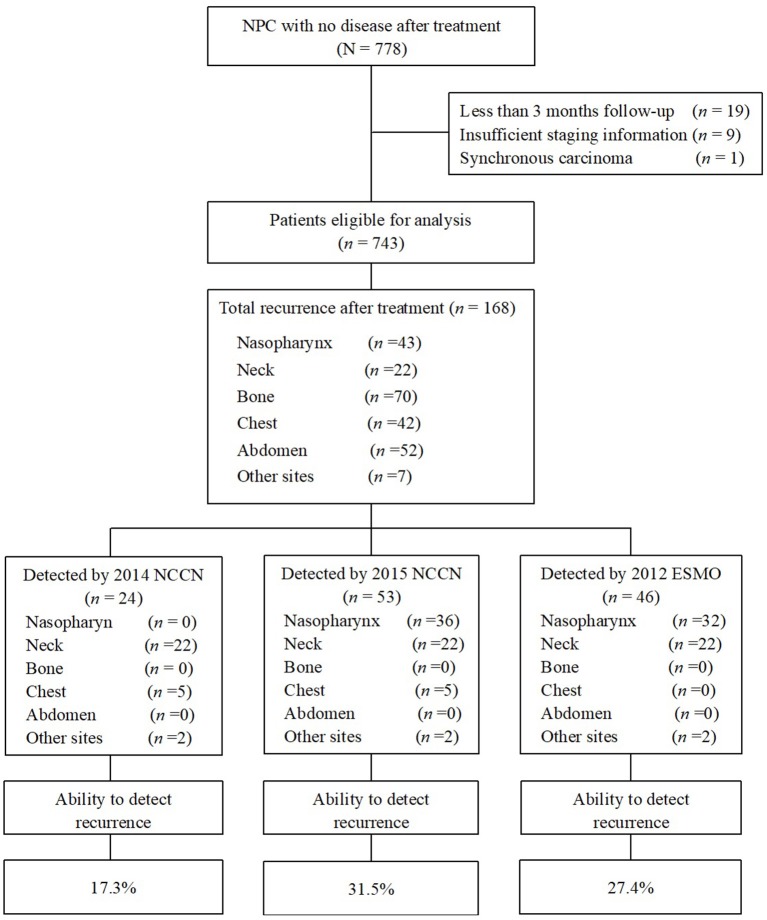
Flowchart of patients enrolled in this study. Ability to detect recurrence (%) = recurrences detected if strictly follow the guidelines/the total number of recurrence after treatment × 100%. NPC, nasopharyngeal carcinoma; NCCN, National Comprehensive Cancer Network; ESMO, European Society for Medical Oncology.

A total of 778 consecutive patients with NPC were enrolled between January 2003 and December 2010. Twenty-nine patients were excluded for the following reasons: fewer than 3 months of follow-up (*n* = 19); insufficient staging information available (*n* = 9); or synchronous carcinoma (*n* = 1), and a total of 749 patients were eligible for analysis (Figure 1). Patient baseline demographic and disease features are summarized in Table 2. There were 580 men and 169 women, with a median age of 43.0 years [interquartile range (IQR) 36–51 years].

**Table 2:** Patient baseline demographic and disease features.

**Characteristics**	**All Patients (*****N*** **=** **749)**	
	**No. of patients**	**%**
**Age (years)**		
≤ 50	553	73.8
>50	196	26.2
**Sex**		
Male	580	77.4
Female	169	22.6
**Pathology type**		
Non-keratinizing carcinoma	744	99.3
Keratinizing squamous cell carcinoma	5	0.7
**Chemotherapy**		
Yes	535	71.4
No	214	28.6
**T category^†^**		
T1–2	317	42.3
T3–4	432	57.7
**N category^†^**		
N0–1	593	79.2
N2–3	156	20.8
**Stage^†^**		
I–II	257	34.3
III–IV	492	65.7

*T, tumor; N, node*.

† *According to the 7th Union for International Cancer Control/American Joint Committee on Cancer staging system*.

### Survival Outcomes

Median post-treatment follow-up for the whole cohort was 100.8 months (IQR 81.4–120.1 months). Of the 749 patients, 168 (22.4%) developed disease recurrence, at a median of 20.6 months (IQR 11.6–38.3 months) after radiotherapy (range 0.8–93.8 months). Among the 168 patients who experienced recurrence, there were 70 bone recurrences (41.7%), 52 abdomen recurrences (31.0%), 43 nasopharynx recurrences (25.6%), 42 chest recurrences (25.0%), 22 neck recurrences (13.1%), and 7 recurrences in other sites (4.2%). A total of 31 patients (18.5%) had recurrence at two or more sites simultaneously. In patients with T3/4 disease, the most common site of recurrence was bone (40.2% bone, 29.1% abdomen, 28.3% nasopharynx, 26.8% chest, 10.2% neck, and 4.7% other). In patients with N2/3 disease, the majority of recurrences were in bone or abdomen (54.2% bone, 33.9% abdomen, 25.4% chest, 23.7% nasopharynx, 8.5% neck, and 5.1% other).

### Performance of Guidelines

When we evaluated the performance of the NCCN and ESMO guidelines to detect recurrences after therapy, we found that the 2015 NCCN surveillance protocol could only find 11.3% of all events. The updated 2018 NCCN T and N stage-adapted protocol improved the overall detection rate to 31.5% (Figure 1). Using a similar T stage-based approach, the 2012 ESMO guidelines enabled detection of 27.4% of all recurrences. Evaluating the ability of 2015 NCCN strategies according to different stage, we found it to be most limited for T1/2 and N2/3 patients, in whom <30% of recurrences would be detected (26.8 and 25.4%, respectively, Table 3). None of the guidelines were able to capture bone or abdominal relapses, because no imaging procedures are recommended for these sites. The 2018 and 2015 NCCN guidelines were able to capture 11.9% of the chest recurrences, with chest imaging recommended for patients with a history of smoking.

**Table 3:** Recurrences captured by the NCCN- and ESMO-prescribed surveillance guideline.

**Recurrences captured**																						
	**Total (*****N*** **=** **168)**		**By clinical stage^†^**												**By recurrence location**							
			**T classification**				**N classification**				**Nasopharynx (*****n*** **=** **43)**		**Neck (*****n*** **=** **22)**		**Bone (*****n*** **=** **70)**		**Chest (*****n*** **=** **42)**		**Abdomen (*****n*** **=** **52)**		**Other (*****n*** **=** **7)**	
			**T1–2 (*****n*** **=** **41)**		**T3–4 (*****n*** **=** **127)**		**N0–1 (*****n*** **=** **109)**		**N2–3 (*****n*** **=** **59)**													
**Guideline**	**No**.	**%**	**No**.	**%**	**No**.	**%**	**No**.	**%**	**No**.	**%**	**No**.	**%**	**No**.	**%**	**No**.	**%**	**No**.	**%**	**No**.	**%**	**No**.	**%**
2018 NCCN	19	11.3	7	17.1	12	9.4	16	14.7	3	5.1	0	0	22	100	0	0	20	47.6	0	0	2	28.6
2015 NCCN	53	31.5	11	26.8	42	33.1	38	34.9	15	25.4	36	83.7	22	100	0	0	20	47.6	0	0	2	28.6
2012 ESMO	46	27.4	7	17.1	39	30.7	34	31.2	12	20.3	32	74.4	22	100	0	0	0	0	0	0	2	28.6

*NCCN, National Comprehensive Cancer Network; ESMO, European Society for Medical Oncology*.

† *According to the 7th Union for International Cancer Control/American Joint Committee on Cancer staging system*.

### Location-Specific Recurrence Patterns

**Figure 2:**
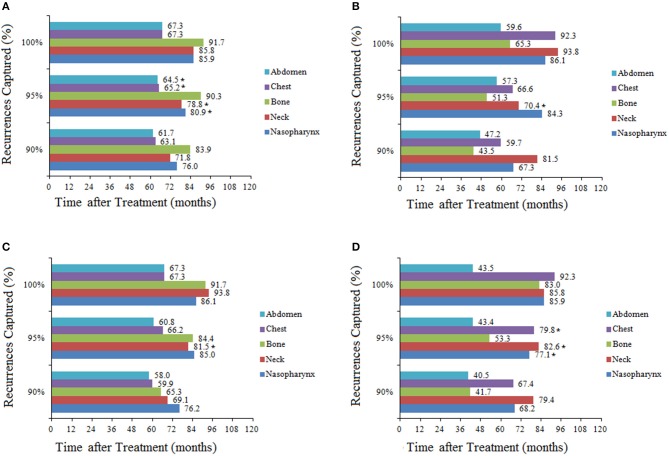
Total duration of surveillance required to capture 90, 95, and 100% of recurrences in patients stratified by stage and recurrence location: **(A)** T1/2; **(B)** T3/4; **(C)** N0/1; **(D)** N2/3. *Estimated duration of surveillance due to there being few recurrences in these groups.

To capture 95% of recurrences, the required surveillance durations were 85.57 and 67.45 months for T1/2 and T3/4 disease (*P* = 0.27), 83.57 and 55.80 months for N0/1 and N2/3 disease, respectively (*P* < 0.001). When location-specific recurrence patterns were incorporated in the analysis for these stages, total surveillance duration of 60 months or longer was required to capture 95% of recurrences, with the exception of bone or abdomen recurrence in T3/4 and N2/3 patients. For example, to capture 95% of recurrences in T1/2 patients, surveillance of the nasopharynx would be required for 80.91 months, neck for 78.82 months, bone for 90.27 months, chest for 65.16 months, and abdomen for 64.50 months (Figure 2). In general, T1/2 and N0/1 patients required longer surveillance than T3/4 or N2/3 for the detection of recurrences in bone or abdomen. For example, to capture 95% of recurrences in N0/1 patients, surveillance of bone would be required for 84.4 months; in N2/3 patients, surveillance of bone would be required for only 53.3 months. The longest surveillance required at any site was bone in T1/2 patients (90.3 months); the shortest surveillance was abdomen in N2/3 patients (43.4 months; Figure 2).

To explore variations in recurrence with time according to stage, we analyzed the 5- and 10-year cumulative recurrence rates at each location (Table 4). Overall, the vast majority of recurrences occurred within the first 5 years, so the 5- and 10-year recurrence rates were similar. There was a consistent trend that the recurrence rate was the lowest in T1/2 group and the highest in N2/3 group for all sites except the neck. The recurrence rate in the neck was low for all stages, with no significant differences (*P* = 0.76 between T1/2 and T3/4, *P* = 0.666 between N0/1 and N2/3, *P* = 0.162 between stage I/II and stage III/IV). The lowest recurrence rate at any site was for the nasopharynx in T1/2 patients, with 5- and 10-year rates of 1.6 and 2.4%, respectively. The highest recurrence rate was observed in bone in patients with N2/3 disease, with 5- and 10-year rates of 22.2 and 22.9%, respectively.

**Table 4:** Recurrence rates at 5 and 10 years by location among different stages.

	**By T classification^†^**			**By N classification^†^**		
**Recurrence location**	**T1–2**	**T3–4**	***P*^#^**	**N0–1**	**N2–3**	***P*^#^**
**Any**			<0.01			<0.01
No. of recurrence	41	127		109	59	
5-year recurrence rate, %	11.7	27.8		16.8	36.8	
10-year recurrence rate, %	13.1	30.2		18.8	38.2	
**Nasopharynx**			<0.01			0.023
No. of recurrence	7	36		29	14	
5-year recurrence rate, %	1.6	7.9		4.2	9.2	
10-year recurrence rate, %	2.4	9.2		5.3	10.1	
**Neck**			0.76			0.666
No. of recurrence	9	13		17	5	
5-year recurrence rate, %	2.7	2.8		2.7	3	
10-year recurrence rate, %	3	4.5		3.1	4	
**Bone**			0.03			<0.01
No. of recurrence	23	51		39	35	
5-year recurrence rate, %	6	12.1		6.1	22.2	
10-year recurrence rate, %	7.5	12.4		6.9	22.9	
**Chest**			<0.01			0.007
No. of recurrence	8	38		30	16	
5-year recurrence rate, %	2.3	8.9		5	10.4	
10-year recurrence rate, %	2.7	9.6		5.3	11.4	
**Abdomen**			0.01			0.002
No. of recurrence	15	41		36	20	
5-year recurrence rate, %	4.5	10		6.1	13.6	
10-year recurrence rate, %	4.9	10		6.3	13.6	
**Other**			0.07			0.166
No. of recurrence	1	7		5	3	
5-year recurrence rate, %	0.3	1		0.7	0.8	
10-year recurrence rate, %	0.3	2		0.9	2.6	

† *According to the 7th Union for International Cancer Control/American Joint Committee on Cancer staging system*.

# *P-values calculated using the Kaplan–Meier method*.

### Medicare Costs Compared Among Guidelines

Then we compared the total Medicare costs of follow-up according to different guidelines, and found that patients followed up under the 2018 NCCN regimen will incur the lowest cost ($1642.66 in 5 years per patients, Table 5). According the 2015 NCCN and 2012 ESMO guidelines, the highest cost (US$4484.69) would be incurred by patients with T3/4 or N2/3 disease because of the requirement for annual baseline imaging. Similarly, due to the baseline imaging recommended by the 2012 ESMO guidelines, a T3/4 patient would incur a greater cost for surveillance than a T1/2 patient. However, to capture 95% of recurrence cases, patients in all groups would incur surveillance costs of ~US$6000, which would be greater than that incurred using the current guidelines.

**Table 5:** Comparison of 2017 medicare costs associated with adhering to the NCCN and ESMO oncologic surveillance schedules and the costs that would be incurred if 95% of all recurrences were captured.

**Surveillance strategy and risk group**	**2017 Total medicare Costs^†^**	**Cost per recurrence case detected**
**2018 NCCN^#^^§^** **(with ability to capture 11.3% of the recurrences)**		
All patients	1,642.66	42,578.64
**2015 NCCN^#^^§^** **(with ability to capture 31.5% of the recurrences)**		
T1–2^*^	2,179.81	62,088.70
T3–4^*^	4,484.69	44,863.95
N0–1^*^	3,254.07	49,595.65
N2–3^*^	4,484.69	46,220.58
**2012 ESMO^#^** **(with ability to capture 27.4% of the recurrences)**		
T1–2^*^	1,642.66	73,329.76
T3–4^*^	3,747.87	40,423.99
**To capture 95% of all recurrences^‡^**		
T1–2^*^	6,264.65	50,338.69
T3–4^*^	5,712.88	19,912.48
N0–1^*^	6,253.04	31,597.83
N2–3^*^	6,237.52	17,187.99

*NCCN, National Comprehensive Cancer Network; ESMO, European Society for Medical Oncology*.

# *The total cost in the first 10 years after treatment were estimated when strictly adhering to surveillance guideline*.

§ *The 2018 and 2015 NCCN recommended annual low-dose chest CT for patients with high risk of lung cancer which represents only 4.82% of the whole patients, so the cost of chest imaging associated adhering to the 2018 and 2015 NCCN was ignored*.

* *According to the 7th edition of the International Union against Cancer/American Joint Committee on Cancer (UICC/AJCC) system*.

† *Estimates based on total costs in dollars incurred by a single patient who has strictly followed and completed the recommended surveillance schedules as outlined in Table 1. H&P exam included both costs of a complete head and neck exam and fiberotic examination*.

‡ *Cost was calculated based on followed estimation: Frequency of H&P exam was similar to that the 2018 and 2015 recommended; baseline imaging included annually head and neck MRI, bone imaging included annually skeletal scintigraphy, chest imaging included annually chest CT, abdomen imaging included annually abdomen CT*.

Regarding the cost to detect per recurrence, we found that US$42,578.64 would be required to detect a recurrence following the 2018 NCCN guidelines. Following the stage-adapted surveillance protocol, detecting a recurrence in a patient with relatively earlier stage disease would cost much more than in a patient with advanced disease because of the lower recurrence rate in the former. For example, the cost per recurrence would be as high as US$62,088.70 in T1/2 patients but only US$44,863.95 in T3/4 patients following the 2015 NCCN recommendations. To capture 95% of all recurrences, the cost per case detected was much less than that incurred using the current guidelines, with US$50,338.69 required to detect a recurrence in patients with T1/2 disease, US$19,912.48 for T3/4, US$31,597.83 for N0/1, and US$17,187.99 for N2/3.

## Discussion

This large-scale study was the first to evaluate NCCN and ESMO follow-up guidelines for NPC. Our results suggested that if these follow-up recommendations from guidelines were strictly followed, it would lead to a large number of missed recurrences. Overall, the 2015 NCCN and 2012 ESMO strategies had an obvious advantage in detecting tumor recurrence because of the individualized recommendations for patients with different stages, yet we found that 69.5 and 72.6% of all recurrences would have been missed, respectively.

Because relapse site and time reflect patterns of recurrence, stratified follow-up according to the characteristics of recurrences can improve the efficiency of follow-up and increase the number of recurrences detected. In our analysis, most recurrences occurred in the first 5 years after treatment and later failures represented <10% of the total. This finding is consistent with previous data. Lee et al. reported that <10% of all local recurrences occurred after 5 years of treatment (8). Therefore, more intensive follow-up during the first 5 years may be justified to detect early locoregional recurrence. In addition, follow-up should continue indefinitely because late recurrences may occur and late recurrences usually have a better prognosis than early recurrences.

The prevailing use of IMRT and concurrent chemoradiotherapy for locoregionally advanced NPC has improved the locoregional control of this disease. As a consequence, distant recurrence has become a predominant pattern of treatment failure (9). However, the current NCCN and ESMO guidelines advocate no regular imaging to detect distant metastatic. The vast majority of early distant recurrences are missed following these guidelines. The most common metastatic sites for NPC include bone, lung and liver (10, 11), which were largely detected through imaging studies (12). Although NPC with distant metastasis was usually considered incurable (13), early detection and treatment of isolated asymptomatic disease could improve survival (14–17). Therefore, early diagnosis of metastatic NPC via routine body imaging instead of symptoms may be of great clinical value.

In our analysis, extending surveillance beyond the current recommendations and integrating routine body imaging in all patients to detect 95% of recurrences would require increased expenditure. The cost per recurrence detected in patients with T1/2 disease was almost three times that in patients with T3/4 disease, due to the better disease control and fewer recurrences in T1/2 patients. This study highlights the importance of developing more reasonable and accurate follow up strategies based on subtypes and risk of relapse, such that patient benefit can be balanced against Medical expense.

We recognize that a limitation of our study was the unstandardized follow-up due to its retrospective design. However, the instituted surveillance protocol was relatively uniform and <3% of patients in this study were lost to follow-up. Our analysis mainly focused on the follow-up period after treatment, while the optimail frequency of radiological examinations remained largely unknown due to the non-standardized follow-up protocol. In this study, the cost of radiological examinations was estimated on annual basis, but we recommend the exploration of more rational and individualized follow-up approaches.

In conclusion, the surveillance guidelines from NCCN and ESMO do not fully capture the recurrence of NCP after radical treatment. Extending surveillance to capture 95% of the recurrence events would lead to higher costs, while the cost per recurrence detected was much less than that incurred following the established guidelines. Detecting recurrence in patients with earlier disease was much more costly than in those with advanced disease. Therefore, the direction of further research was to identify personalized review strategies to balance the benefits of patients with medical costs.

## Data Availability Statement

All datasets generated for this study are included in the article/supplementary material.

## Ethics Statement

The study was approved by the institutional review board of Sun Yat-sen University Cancer Center.

## Author Contributions

We declare that all authors are qualified. G-QZ, J-WL, JM, and YS had substantial contributions to the conception and design of the work, drafting the work, and revising it critically for important intellectual content or the acquisition. LT, Y-PM, and RG analyzed the data for the work. All authors provided approval for publication of the content. YS agreed to be accountable for all aspects of the work in ensuring that questions related to the accuracy or integrity of any part of the work are appropriately investigated and resolved.

### Conflict of Interest

The authors declare that the research was conducted in the absence of any commercial or financial relationships that could be construed as a potential conflict of interest.
